# Controllable Synthesis of Three-Dimensional Chiral Au Nanoflowers Induced by Cysteine with Excellent Biocompatible Properties

**DOI:** 10.3390/nano14242040

**Published:** 2024-12-19

**Authors:** Shengmiao Liu, Jianhao Zhang, Wenjing Yan

**Affiliations:** 1 College of Food Science and Technology, Nanjing Agricultural University, Nanjing 210095, China; 2022108071@stu.njau.edu.cn (S.L.); nau_zjh@njau.edu.cn (J.Z.); 2 Key Laboratory of Food Contact Materials Safety, State Administration for Market Regulation, Nanjing 210095, China

**Keywords:** chiral nanomaterials, cysteine, *g*-factor, optical properties, biocompatibility

## Abstract

Chiral molecules are ubiquitous in nature and biological systems, where the unique optical and physical properties of chiral nanoparticles are closely linked to their shapes. Synthesizing chiral plasmonic nanomaterials with precise structures and tunable sizes is essential for exploring their applications. This study presents a method for growing three-dimensional chiral gold nanoflowers (Au NFs) derived from trisoctahedral (TOH) nanocrystals using D-cysteine and L-cysteine as chiral inducers. By employing a two-step seed-mediated growth approach, stable chiral Au nanoparticles with customizable sizes, shapes, and optical properties were produced by adjusting the Au nanosphere (Au NP) seed concentration and cysteine dosage. These nanoparticles exhibited optical activity in both the visible and near-infrared regions, with a maximum anisotropy factor (*g*-factor) of 0.024. Furthermore, the PEG-modified chiral Au NFs demonstrated excellent biocompatibility. This approach provides a precise method for geometrically controlling the design of three-dimensional chiral nanomaterials, holding great potential for biomedical applications.

## 1. Introduction

Chirality, the property of an object that prevents it from being superimposed on its mirror image, is a defining feature in many biological macromolecules [1]. This structural asymmetry plays a crucial role in biology and chemistry, primarily by interacting with polarized light to produce unique optical phenomena. It selectively absorbs circularly polarized light, either left-handed (LCP) or right-handed (RCP), resulting in a distinct absorption spectrum known as circular dichroism (CD) [2,3]. However, most biomolecules, including DNA, amino acids, and peptides, exhibit chirality responses only in the ultraviolet range, typically yielding relatively weak chiral optical activities [4].

In recent years, chiral plasmonic nanostructures have attracted considerable attention due to their unique optical properties and ability to enhance nanoscale light–matter interactions [5]. Their optical characteristics, which are dependent on morphology and size, allow for the tuning of chiral optical signals in the visible and near-infrared regions, resulting in significant amplification. The local surface plasmon resonance (LSPR) effect in these structures generates a strong chirality signal at the resonance frequency, thus improving the sensitivity and precision of chiral detection [6,7]. These characteristics make chiral plasmonic nanostructures ideal candidates for various biomedical applications, including biosensing [8,9], photothermal therapy [10,11], drug delivery [12], and targeted imaging [13].

Among the various methods currently available for preparing chiral plasmonic nanostructures, seed-mediated growth using chiral ligands has emerged as an effective strategy for producing enantiomeric nanoparticles with significant chirality [14,15]. This technique reduces a noble metal precursor to a non-chiral seed and produces a chiral morphology by controlling the reaction conditions. Using this approach, researchers have synthesized a variety of chiral gold nanostructures such as 432-symmetric helical nanoparticles [16], twisted nanorods [17,18], chiral starfruit [19], nanoplates [20], and nanopropellers [21]. Other methods, including chiral micellar templates [22], circularly polarized light (CPL) irradiation [23], and electron-beam lithography [24], have also shown promise in the synthesis of chiral nanostructures.

Despite these advancements, achieving precise control over the morphology, size, and chirality of chiral plasmonic nanoparticles remains challenging. The methods described above often lack flexibility and scalability, and they are time-consuming. In particular, rapidly synthesizing three-dimensional chiral nanostructures with high optical activity in solution-based reactions remains difficult, highlighting the need for innovative and adaptable synthesis strategies.

In this work, we propose a two-step seed-mediated growth method to generate stable, size- and shape-controllable chiral Au nanoparticles by systematically tuning their chiral structures through precise adjustment of chemical parameters. Gold (Au) is an ideal material for synthesizing chiral plasmonic nanoparticles due to its remarkable optical properties, including LSPR [25,26], as well as its chemical stability and exceptional biocompatibility. Cysteine (Cys), a naturally occurring chiral amino acid, serves as an ideal chiral inducer for nanoparticle synthesis due to its inherent chirality and strong thiol–gold (Au–S) interactions [27]. Cys selectively adsorbs onto the high-Miller-index facets of Au seeds, directing anisotropic growth and inducing chirality. The combination of Au and Cys offers a robust and efficient strategy for the design and synthesis of chiral nanostructures.

In our approach, the first step involves synthesizing Au trisoctahedral (Au TOH) seeds from spherical particles at varying concentrations to produce intermediate shapes that serve as starting points for chiral evolution. These Au TOH seeds are then further grown in a second solution, where Cys is used as a shape-directed additive to break the symmetry of the TOH seeds and induce chirality in the nanoparticles. Taking advantage of the unique crystallographic and geometrical features of the TOH structure, this approach enables the creation of three-dimensional chiral gold nanoflowers (abbreviated as D-Au NFs and L-Au NFs). Cys not only determines the direction of chiral growth but also controls the degree of chiral distortion and growth kinetics. Notably, the second growth step is completed in just 20 min, and the resulting nanoparticles exhibit a significant anisotropy factor (*g*-factor) and good biocompatibility through PEG modification. This strategy provides a practical approach to the creation of customizable chiral nanomaterials with promising applications in the biomedical field.

## 2. Materials and Methods

### 2.1. Materials and Reagents

Chloroauric acid (HAuCl_4_, 99%), D-Cysteine (D-Cys, 99%), and L-Cysteine (L-Cys, 99%) were purchased from Sigma-Aldrich, St. Louis, MO, USA. Ascorbic acid (AA, 99%) and Ethylenediaminetetraacetic acid disodium salt (EDTA-2Na, 98%) were obtained from Solarbio, Beijing, China. Sodium borohydride (NaBH_4_, >98%) was obtained from Aladdin, Shanghai, China. Cetyltrimethylammonium bromide (CTAB, 99%) and Cetyltrimethylammonium Chloride (CTAC, 99%) were obtained from Macklin, Shanghai, China. Polyethylene Glycol (HS-PEG_5000_-NH_2_, >95%) was purchased from Ponsure Biological (Shanghai, China). All glassware used in the synthesis was soaked in aqua regia overnight, thoroughly washed, and then dried before use. Ultrapure water (18.2 MΩ), obtained from a Milli-Q ultrapure water apparatus, was used for all experiments. All chemicals were used without further purification.

### 2.2. Preparation of Au Nanospheres (Au NPs)

The synthesis of Au NPs was based on previously reported methods with modifications [28]. Au clusters were prepared by mixing 0.6 mL of NaBH_4_ (10 mM, prepared in ice water), 5 mL of aqueous HAuCl_4_ (0.25 mM), and 5 mL of aqueous CTAB (100 mM). The mixture was maintained at 37 °C for 3 h to allow the decomposition of NaBH_4_. Subsequently, 100 μL of Au clusters were added to a growth solution containing HAuCl_4_ (0.5 mM, 2 mL), CTAC (200 mM, 2 mL), and AA (100 mM, 1.5 mL). After 10 min, the solution turned pink, indicating the formation of the Au NP seed solution, which was then purified by centrifugation at 15,000 rpm for 15 min. The resulting crystals were washed once, redispersed in 1 mM CTAC solution, and then set aside.

### 2.3. Synthesis of Au TOH Seeds

The Au NP seeds were diluted to various concentrations (38.8 μg/mL, 31.4 μg/mL, 26.4 μg/mL, 22.8 μg/mL, 20.0 μg/mL, and 17.8 μg/mL) and introduced into growth solutions containing CTAC (100 mM, 4 mL), Au-EDTA (0.5 mM, 3 mL), and AA (100 mM, 260 μL). The Au-EDTA precursor was synthesized by reacting EDTA-2Na with HAuCl_4_ in equal molar amounts (10 mM), and the resulting solution was diluted to a final Au(III) concentration of 0.5 mM. The reaction was performed on an oscillator at 10 °C and 250 rpm for 30 min. The resulting Au TOHs were washed by centrifugation (5000 rpm for 5 min), dispersed in 1 mM CTAB, and then set aside.

### 2.4. Synthesis of Chiral Au NFs

A volume of 100 μL of HAuCl_4_ (10 mM) was dispersed in 1.6 mL of CTAB (10 mM) solution and diluted with 8 mL of ultrapure water. Next, 0.95 mL AA (100 mM) and a different volume of 0.075 mM D-Cys or L-Cys were added. Finally, 0.1 mL Au TOH seeds were added. The reaction was carried out at 37 °C for 30 min. The chiral material was washed by centrifugation three times (4000 rpm, 3 min), resuspended in ultrapure water, and then stored at 4 °C.

### 2.5. Calculation of g-Factor Values

The *g*-factor was calculated using the following formula:

(1) $$g-\mathrm{factor}=\frac{circular\;dichroism(in\;mdeg)}{32980\times absorbance\;value}$$

where circular dichroism values were derived from CD spectra and absorbance values were obtained from UV–Vis spectra.

### 2.6. Polyethylene Glycol (PEG) Coating

Initially, the chiral Au NF solution was taken and centrifuged, and then the precipitate was gently stirred into 1 mL of HS-PEG_5000_-NH_2_ solution at room temperature. After 24 h, it was recollected by centrifugation and resuspended in ultrapure water. Finally, the precipitate was stored in the refrigerator at 4 °C as a backup.

### 2.7. Cytotoxicity Analysis

MTT Assay: HeLa cells were seeded in 96-well plates and cultured for 24 h. Afterwards, the culture medium was removed, and 150 μL of chiral nanostructures at varying concentrations were added to each well, with PBS as the control. The samples were then incubated at 37 °C for 24 h. Following incubation, 50 μL of MTT reagent was added to each well and the plates were incubated for an additional 4 h. The absorbance at 490 nm was measured using a BioTek enzyme marker to assess cell viability.

Fluorescence staining: Cell suspensions were added to pre-incubated cell crawls in well plates, allowing the cells to adhere for 24 h. Afterwards, the culture medium was aspirated and replaced with fresh medium containing chiral nanostructures. The cells were incubated for 24 h, followed by three washes with PBS (pH = 7.4) at 37 °C. FITC-labeled Ghost Pen Cyclic Peptide solution was then added dropwise to the cells, and the samples were incubated for 40 min at 37 °C to stain the cytoskeleton. After incubation, the cells were washed three times with PBS. To stain the nuclei, a drop of DAPI dye was added to the cell crawls and incubated for 30 s. The cells were then washed again with PBS. Finally, the samples were sealed with an anti-fluorescence burst sealer and observed under a fluorescence microscope to assess cell morphology.

### 2.8. Characterizations

The absorption spectra were recorded with an ultraviolet–visible ray (UV–Vis) spectrophotometer (Shimadzu, Kyoto, Japan). The circular dichroism (CD) spectra were obtained using a J-1500 spectropolarimeter instrument (JASCO, Tokyo, Japan) with a 1 mm quartz CD cuvette. The CD spectrum in the 400–1000 nm wavelength range was recorded at 298 K under continuous nitrogen-washing protection. The Au content of the samples was determined using an inductively coupled plasma mass spectrometer (ICP-MS, Agilent 7850, Santa Clara, CA, USA). Scanning electron microscopy (SEM) images were taken using a JEOL JSM-6700F microscope (JEOL, Tokyo, Japan) operating at 15 kV. The products were characterized by transmission electron microscopy (TEM) (Tecnai 12, FEI, Hillsboro, OR, USA). A zeta potential analyzer (Nano ZS90, Zetasizer, Malvern, UK) was used to estimate the change in zeta potential (ζ) of chiral Au NFs before and after modification of PEG.

## 3. Results

### 3.1. Synthesis of Au TOHs

**Figure 1:**
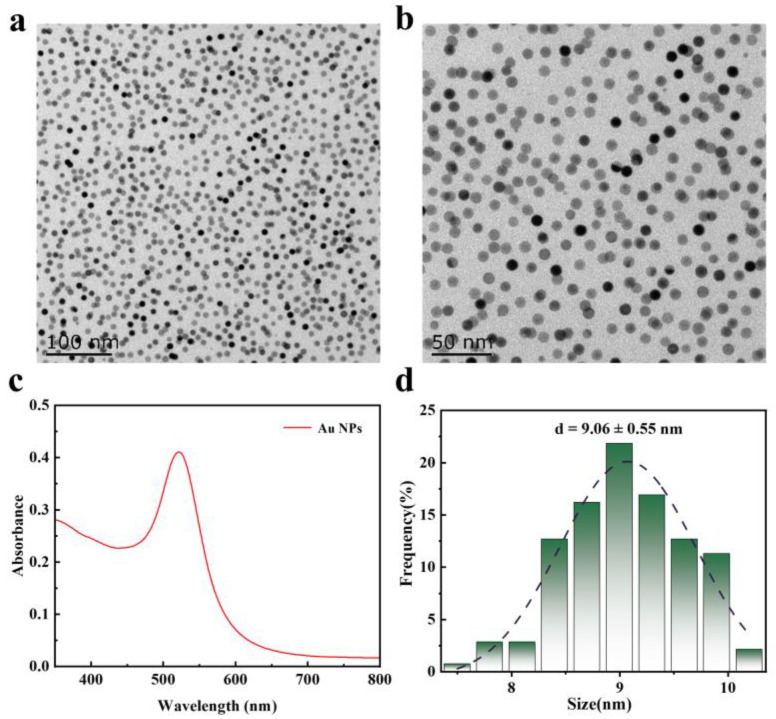
Characterization of the Au NP seeds. (**a**,**b**) Representative TEM images; (**c**) UV–Vis spectra, and (**d**) statistical analysis of particle size of Au NP seeds.

**Figure 2:**
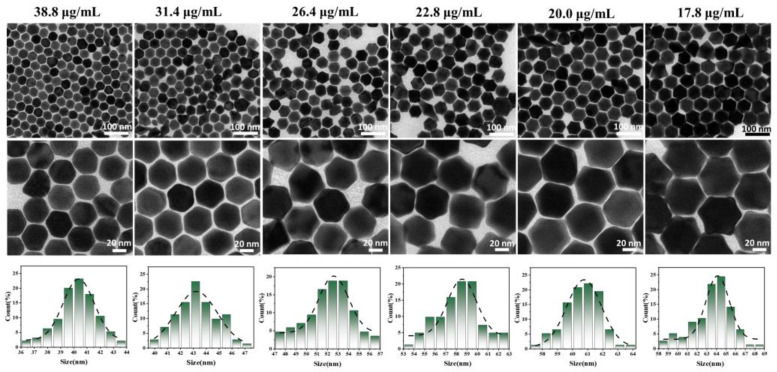
TEM images and particle size distribution of Au TOHs synthesized with Au NP seeds at various concentrations.

We first synthesized uniformly shaped and sized Au NP seeds (Figure 1a,b). UV–Vis spectra indicated that the corresponding LSPR peaks of the Au NPs were located at 521 nm (Figure 1c). The particle size of the Au NPs was determined through statistical analysis of TEM images, with an average particle size of approximately 9 nm (Figure 1d). The morphology and size of Au TOHs synthesized in the presence of different Au NP seed concentrations are shown in Figure 2. During seed-mediated growth, other reaction parameters are kept constant, seed concentration is used as the only variable, and the size of the nanocrystals depends mainly on the average number of atoms that can be deposited on individual crystal seeds. Therefore, the higher the number of seeds involved, the smaller the size of the formed product. TEM observations showed that the size (diagonal length) of the Au TOHs increased with the decrease in the Au NP seed concentration, and the average particle size increased from 40.3 nm to 63.6 nm when the Au NP seed concentration was reduced from 38.8 μg/mL to 17.8 μg/mL (Figure 2, Table 1).

**Figure 3:**
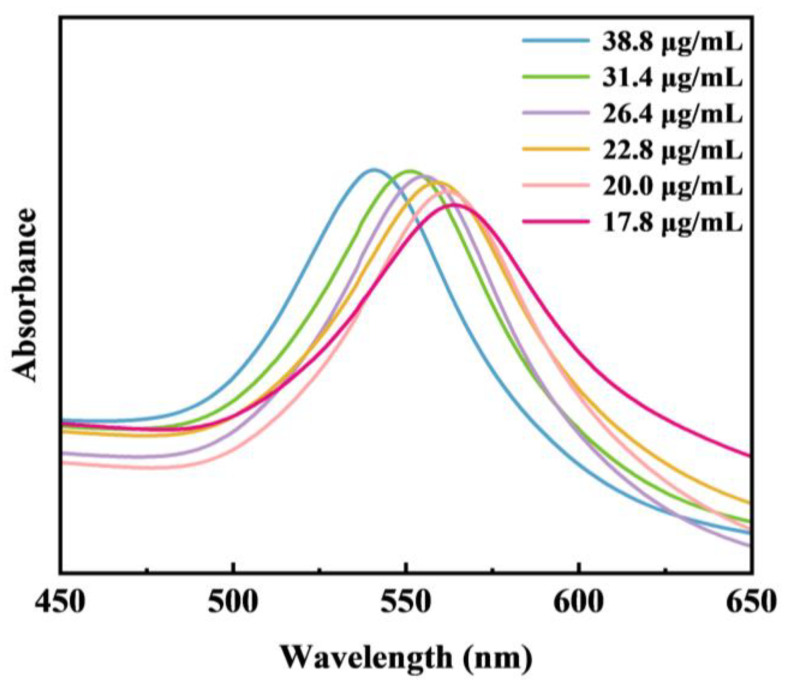
UV–Vis spectra of Au TOHs synthesized with Au NP seeds at different concentrations.

Interestingly, the smaller the Au NP seed concentration, the more prominent the tip of the synthesized Au TOH nanoparticles, and the larger the dihedral angle. This change can be attributed to rearranging the atoms on the corresponding faces, where the number of atoms on the {111} faces gradually exceeds the number of atoms on the {110} faces on a stepwise basis [28,29]. This corresponds to the optical measurements shown in Figure 3: the corresponding LSPR peaks of Au TOHs are monotonically redshifted from 541 nm to 564 nm. This may be because the increased dihedral angle makes the nanocrystals more morphologically anisotropic, which is more favorable for the redshift of the surface plasmon resonance peaks. This result suggests that it is feasible to control the size, dihedral angle, and optical properties of Au TOHs by controlling the amount of Au NP seeds.

### 3.2. Effect of AuNP Seed Concentration on Chiral Au NFs

**Figure 4:**
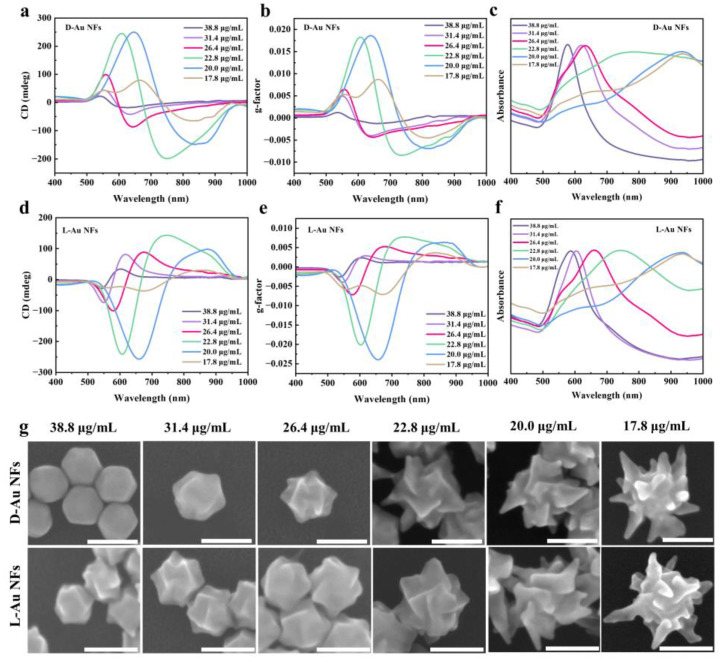
SEM images of chiral Au NFs synthesized with different concentrations of Au NP seeds and corresponding optical properties. (**a**,**d**) CD spectra, (**b**,**e**) *g*-factor spectra, (**c**,**f**) UV–Vis spectra, and (**g**) SEM images of chiral Au NFs (the amount of Cys was 30 μL). Scale bars: 100 nm.

The crystal morphology of the initial seeds during chiral growth is a critical factor in determining the final shape of the chiral nanocrystals [30]. Therefore, we used Au TOHs, synthesized from different concentrations of Au NP seeds, as intermediates to provide diverse starting points for chiral evolution. During the growth process, in the presence of the CTAB surfactant and using D-Cys or L-Cys as surface inducers, gold (III) chloride is reduced by AA, enabling growth from TOH seeds. This process breaks the symmetry of the TOH seeds, resulting in Au nanocrystals with opposite chiral morphologies. CTAB facilitates the transfer of chirality from the chiral molecules to the nanocrystals, suppressing uncontrolled overgrowth on non-chiral crystal planes and promoting the formation of well-defined chiral structures. Under otherwise identical synthesis conditions, the chiral crystal shapes and optical properties vary with the concentration of Au NP seeds, as shown in Figure 4. L-Au NFs and D-Au NFs exhibit clockwise and counterclockwise rotations, respectively, resulting in strong geometric and optical asymmetry. The chiral-specific binding affinity between the R/S kink sites on the crystal surface and D-Cys or L-Cys induces asymmetric growth [31]. Nanoparticles with opposite chiral ligands exhibit similar UV–Vis spectra and nearly perfect mirror-symmetric CD spectra (Figure 4a–f).

The morphological evolution of D-Au NF and L-Au NF nanoparticles at varying Au NP seed concentrations was analyzed using scanning electron microscopy (SEM) images (Figure 4g), revealing transitions from compact to more intricate structures. Notably, these morphological changes correlate with variations in light–matter interactions, influencing the nanoparticles’ plasmonic and chiroptical properties. The chiral structures develop from the apex of the TOH seeds, with higher seed concentrations (38.8 μg/mL) producing flat, compact TOH-like shapes. As the seed concentration decreases, the chiral twist becomes more pronounced. At a concentration of 22.8 μg/mL, the particles develop distinctly curved, petal-like surfaces with sharper edges. At 20.0 μg/mL, the particles adopt a more open, four-bladed windmill-like shape with prominent outward projections. At the lowest concentration of 17.8 μg/mL, the particles become highly branched, resembling a sea urchin structure, with sizes increasing from approximately 85 nm to 250 nm. The evolution from compact to branched structures can be attributed to changes in growth kinetics, where lower seed concentrations lead to more concentrated growth at active sites, promoting anisotropic growth along high-Miller-index facets, as reflected in their optical properties [32] (Figure 4a–f). As the Au NP seed concentration decreases, the CD peak wavelength (λ_max) of these chiral nanocrystals gradually redshifts from approximately 540 nm to 670 nm, consistent with the increasing complexity of their geometries. The CD signal intensity first increases and then decreases, peaking at 20.0 μg/mL, with a *g*-factor of 0.019 for D-Au NFs and −0.024 for L-Au NFs. This behavior likely reflects the interplay between chiral molecular interactions and crystal geometry, where an optimal seed concentration facilitates molecular adsorption and orientation, enhancing chiroptical activity. Additionally, a significant redshift in the LSPR peak (from 680 nm to 938 nm) occurs, which is attributed to the increased anisotropy of the nanoparticles. This redshift enables tunable adjustments from visible to near-infrared wavelengths, making these nanoparticles highly relevant for bio-imaging and photothermal therapy applications.

The differences in optical properties between D-Au NFs and L-Au NFs structures at the same Au seed concentration merit further discussion. Under identical conditions, D- and L-type chiral nanoparticles exhibit different levels of optical activity, as shown in the CD spectra and *g*-factor values in Figure 4a–f. This disparity may stem from slight differences in molecular alignment and lattice twist direction, which could influence how Cys molecules interact with the surface and affect the resulting optical chirality. These subtle variations highlight the inherent complexity of chiral induction and suggest that even minor structural differences can lead to significant optical property variations.

### 3.3. Effect of Cys Concentration on Chiral Au NFs

**Figure 5:**
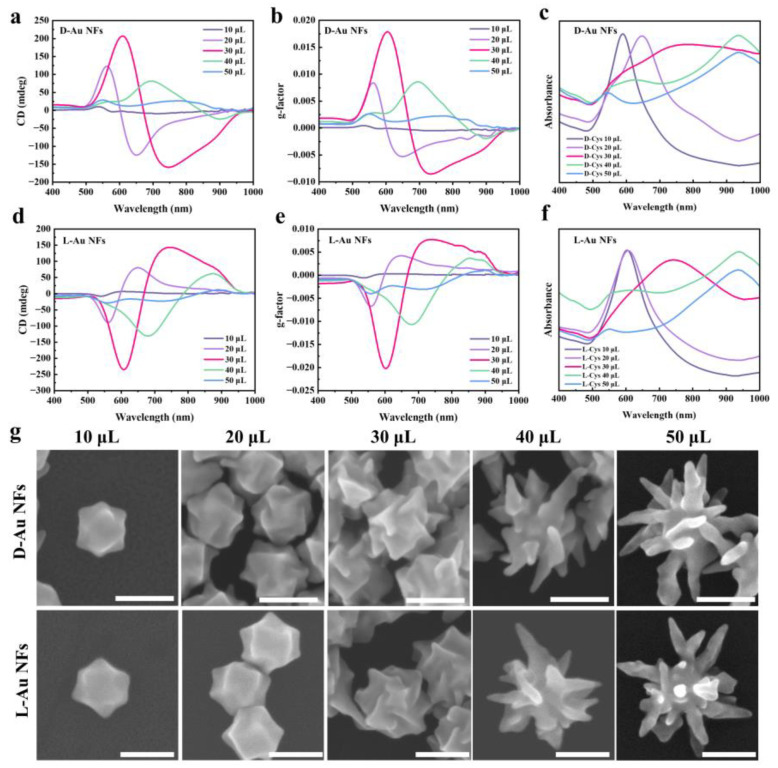
SEM images of chiral Au NFs synthesized with different volumes of Cys and corresponding optical properties. (**a**,**d**) CD spectra, (**b**,**e**) *g*-factor spectra, (**c**,**f**) UV–Vis spectra, and (**g**) SEM images of chiral Au NFs (the concentration of Au NP seeds was 22.8 μg/mL). Scale bars: 100 nm.

In addition to the effect of seed concentration, the amount of Cys used as a molecular chiral inducer plays a crucial role in defining the chiral structure and optical activity of nanoparticles. The strong Au-S binding energy influences Au atom deposition during growth, promoting high-Miller-index facets that enhance the nanoparticles’ chiral character. Cys molecules preferentially adsorb onto stepped Au surfaces, binding to bridge sites along step edges, thereby facilitating directional growth and promoting chiral twisting [33]. As shown in Figure 5g, with the addition of 10 μL of Cys solution, the nanoparticles display a compact, symmetric polyhedral shape with smooth facets and an approximate size of 105 nm. As the concentration of Cys increases, the triangular pyramid structure undergoes a marked rotation, likely due to Au-S interactions directing Au atom deposition onto high-index facets. As the volumes of Cys solution increase to 20 μL, the nanoparticles grow to about 115 nm, and the chiral features become more pronounced. At 30 μL, the nanoparticles reach approximately 150 nm, developing well-defined chiral structures with pronounced curvatures and deeper concavities. This suggests that this concentration allows for optimal Cys adsorption and interaction at key growth sites. In the presence of more Cys solution, such as 50 μL, the nanoparticles exhibit a highly branched, star-like shape, growing to approximately 260 nm. This excessive growth indicates that higher Cys levels may lead to overgrowth at the crystal edges, reducing distinct chiral features.

The morphological transitions observed in Figure 5g closely correlate with the changes in optical properties shown in Figure 5a–f. As shown in Figure 5a,d, the CD spectra indicate that the CD signal begins to enhance with increasing Cys concentration, peaks at 30 μL, and then declines. This trend is also reflected in the *g*-factor values (Figure 5b,e), where the chiral nanoparticles show the highest optical chirality at 30 μL, with a *g*-factor of 0.018 and −0.020 for D-Au NFs and L-Au NFs, respectively. These results are consistent with the well-defined twisted surfaces observed at this concentration, where optimal molecular alignment enhances the chiroptical activity. At 40 μL and 50 μL, the *g*-factor values decrease due to surface saturation caused by excessive Cys. This saturation reduces selective interactions with homochiral facets, resulting in less defined chiral structures and negatively impacting chiral optical properties.

Additionally, the UV–Vis spectra in Figure 5c,f reveal a significant redshift in the LSPR peak, shifting from approximately 595 nm at lower Cys concentrations to 935 nm at higher concentrations. This redshift suggests an increase in particle anisotropy and size, as corroborated by the SEM images in Figure 5g. The LSPR response becomes more pronounced as the chiral nanostructures evolve from compact polyhedral shapes to highly branched, star-like structures. This phenomenon underscores the correlation between morphology and optical properties, demonstrating that Cys-induced anisotropic growth plays a pivotal role in tuning the plasmonic response of the nanoparticles.

### 3.4. Biocompatibility Studies

The excellent biocompatibility of nanostructures is an important issue for their further biomedical applications. CTAB is a widely used surfactant in the synthesis of nanostructures due to its ability to stabilize particle morphology and control the anisotropic growth of nanoparticles [34,35,36]. However, CTAB-coated nanoparticles are known for their high cytotoxicity, which poses challenges for direct biomedical applications [37]. To address this limitation, we modified the surface of the chiral Au NFs with PEG, which replaced the CTAB molecules through the formation of strong covalent Au–S bonds between the Au atoms and the sulfhydryl groups of PEG. This modification not only effectively removed CTAB from the nanoparticle surface but also significantly improved the biocompatibility of the nanoparticles.

**Figure 6:**
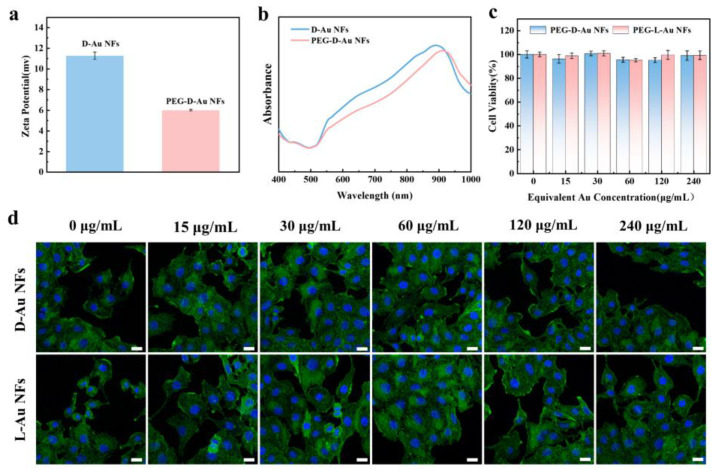
Cytotoxicity analysis of chiral Au NFs. (**a**) Zeta potential and (**b**) UV–Vis spectra of D-Au NFs before and after modification with PEG. (**c**) Cell viability of Hela cells after treatment by PEG-D-Au NFs and PEG-L-Au NFs. (**d**) The fluorescent images of Hela cells after being cocultured with chiral Au NFs at different concentrations, with F-actin stained with FITC (green) and nuclei stained with DAPI (blue). Scale bars: 20 μm.

The zeta potentials of the chiral Au NF nanoparticles before and after PEG modification are shown in Figure 6a, where the potential of CTAB-coated D-Au NFs is about +11.3 mV, while that of D-Au NFs nanoparticles after PEG modification decreases to about +5.9 mV. Meanwhile, the change in absorption spectrograms also proves the successful modification of PEG in Figure 6b. After PEG modification on the surface of D-Au NF nanoparticles, the absorption peak of the nanoparticles was red-shifted by about 22 nm.

As shown in Figure 6c, the concentration of PEG–chiral Au NF nanoparticles increased from 0 to 240 μg/mL, and cell viability remained at a consistently high level (>95%). Moreover, the cell morphology images in Figure 6d show that even in the presence of high concentrations of PEG–chiral Au NF nanoparticles, the number and morphology of the cells did not change, and they still had unique synaptic and branching structures. This observation confirms that PEG-modified nanoparticles do not induce stress responses or alter cell structure, further demonstrating the excellent biocompatibility of synthetic nanoparticles.

## 4. Conclusions

In summary, our study demonstrates a successful two-step seed-mediated growth method for synthesizing chiral Au NF nanoparticles with tunable morphologies and optical properties by controlling seed and Cys concentration. Cys molecules play a critical role in determining chirality, degree of twist, and chiral growth dynamics, while the TOH shape of the intermediate seeds is a key factor in chiral structural evolution. PEG modification, achieved through covalent Au–S bonding, effectively replaces CTAB, endowing PEG–chiral Au NFs with excellent biocompatibility, making them suitable for potential biomedical applications. The optimized CD and LSPR responses underscore their potential in biosensing, targeted drug delivery, photothermal therapy, and real-time bio-imaging.

Although the MTT assay used in this study provides valuable insights into the biocompatibility and cell viability of the chiral Au NFs, it is important to acknowledge that it is a general assay and may not capture all the detailed mechanisms involved. While more specific techniques, such as PCR assays, could provide further insights, the MTT assay remains a suitable approach for evaluating the overall biocompatibility within the scope of this study.

The synthesis method presented in this study contributes to the broader field of chiral nanomaterials by providing a reliable approach to structural and optical tuning, which could be extended to other types of nanoparticles and applied to a wide range of scientific and industrial fields.

## Figures and Tables

**Table 1:** Summary of particle sizes for Au TOHs synthesized with Au NP seeds at various concentrations.

Concentration (μg/mL)	Mean Size (d, nm)	Standard Deviation (σ, nm)
38.8	40.3	±1.5
31.4	43.3	±1.7
26.4	52.1	±2.1
22.8	58.3	±2.0
20.0	60.6	±1.3
17.8	63.6	±2.1

## Data Availability

The data presented in this study are available on request from the corresponding author.
